# Supplementary material for: Multi-Omic Approach to Identify Phenotypic Modifiers Underlying Cerebral Demyelination in X-Linked Adrenoleukodystrophy
Source: Front Cell Dev Biol. 2020 Jun 25;8:520. doi: 10.3389/fcell.2020.00520 (PMC7330173; doi:10.3389/fcell.2020.00520)
Supplement: Supplementary file 2 [file Data_Sheet_1.docx]

Supplementary Material

# Overview

- [Supplementary Figures](#_Supplementary_Figures)
- [Supplementary Tables](#_Supplementary_Tables)
- [Supplementary Results](#_Supplementary_Results)
- [Supplementary Methods](#_Supplementary_Methods)
- [Supplementary References](#_Supplementary_References)

# Supplementary Figures


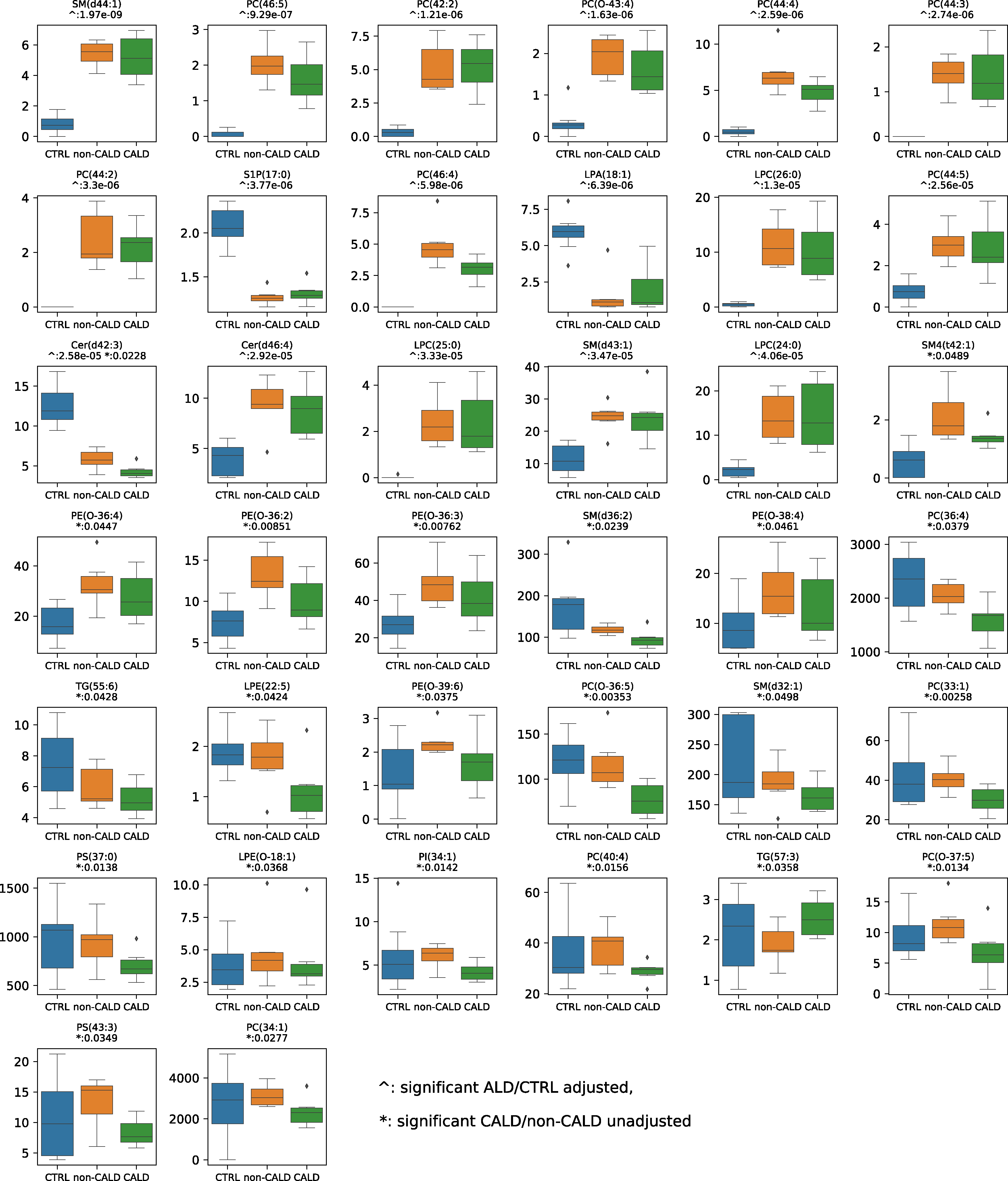


**Supplementary Figure 1.** Boxplots for the lipids showing significant differential abundance in both the comparison between control and ALD, as well as the lipids which did not reach significance but passed p-value <0.05 between non-CALD and CALD. Values are lipid abundances measured on LCMS.


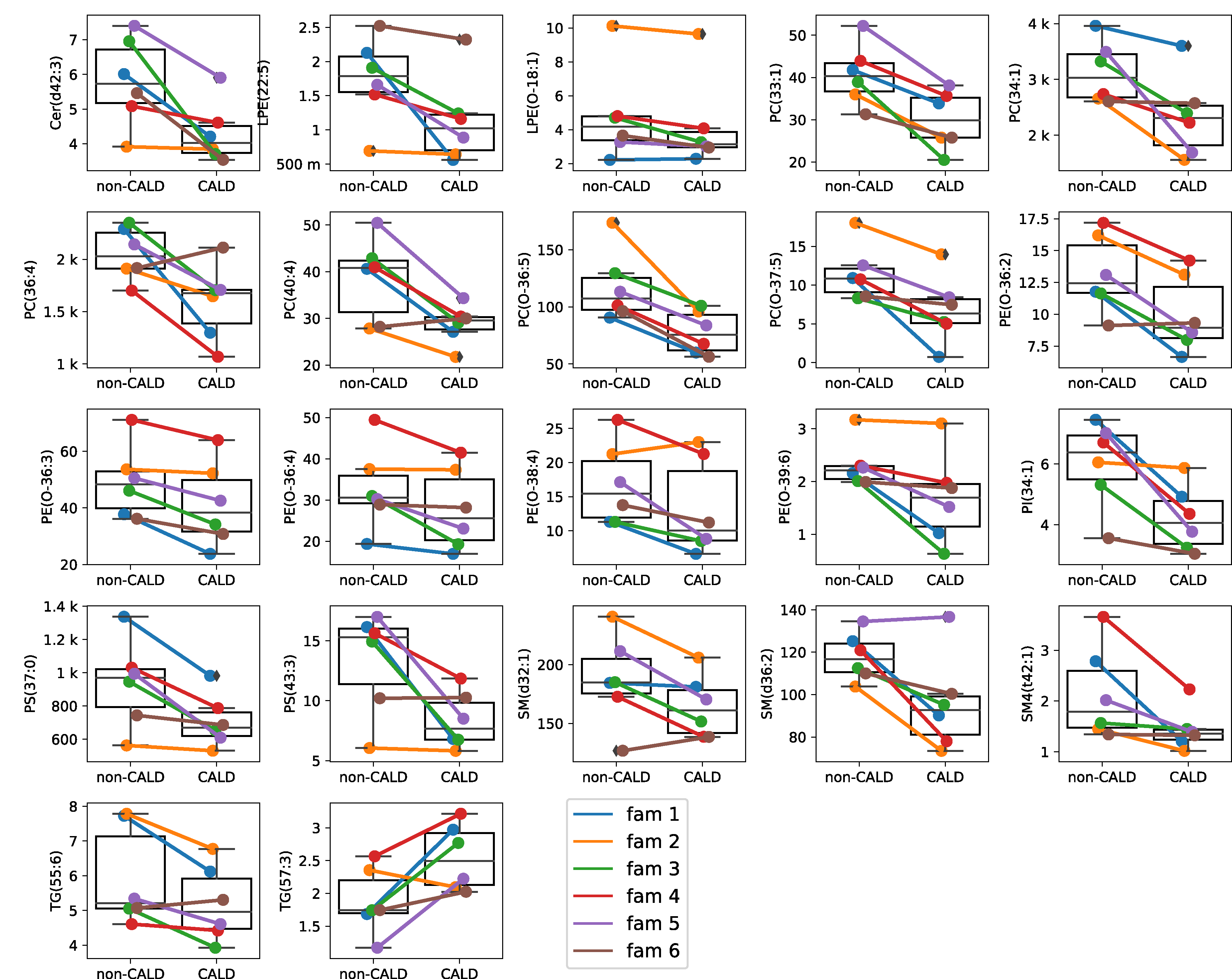


**Supplementary Figure 2.**  Annotated boxplots for significant CALD vs. non-CALD lipids.

Each lipid is drawn as a boxplot for non-CALD and CALD patients, with each family pair annotated as a connected line with points representing the normalized lipid measurement. These hits are selected based on passing a significance threshold (unadjusted p-value < 0.05). Values on y-axis represent relative lipid intensity.


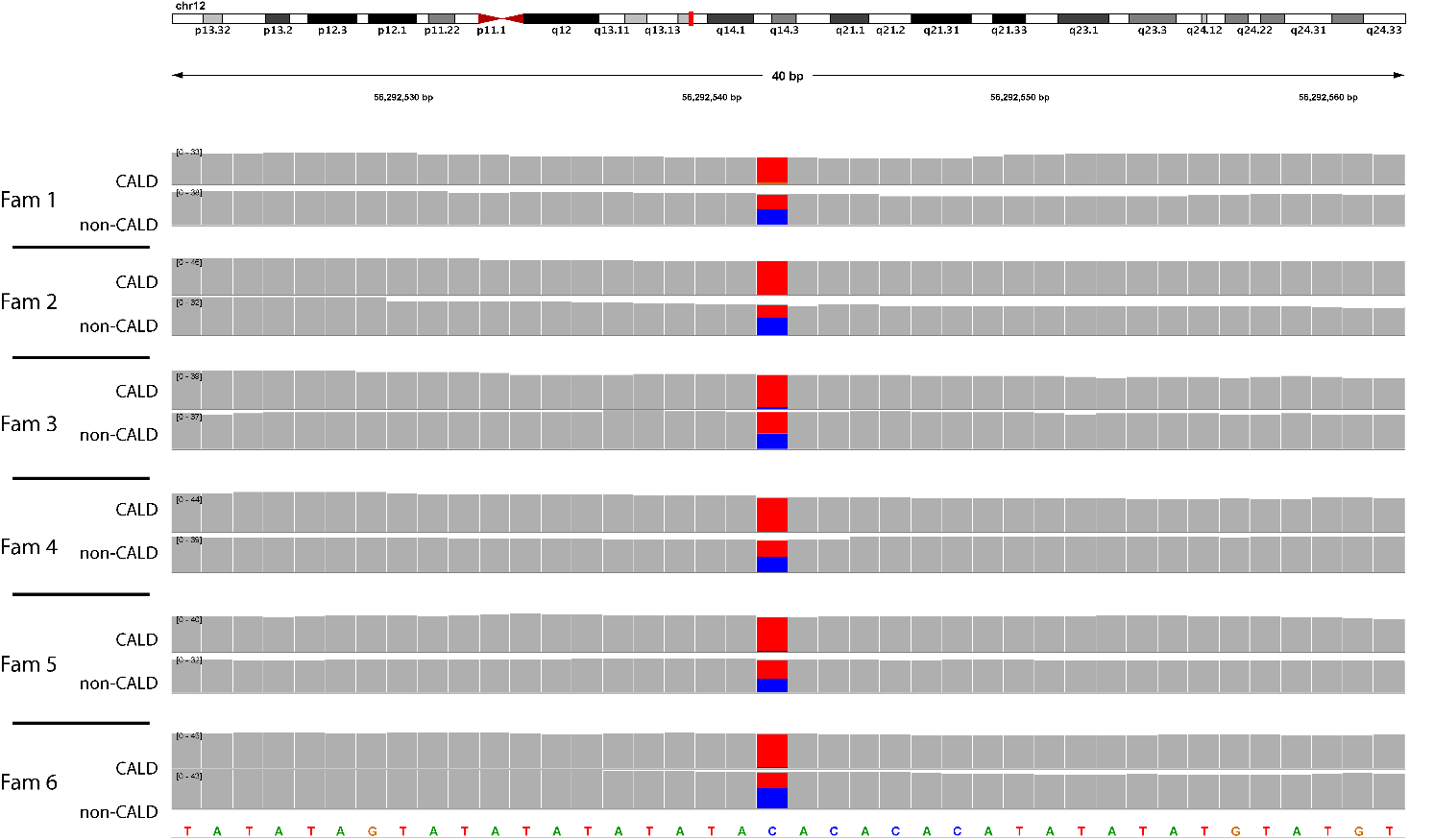


**Supplementary Figure 3.** Segregating alleles downstream of *WIBG* (*PYM1*) depicted in an

IGV snapshot of the mapped read coverages for each of the brother pairs. The specific SNV, upstream of the *WIBG*/*PYM1* gene, is centered in the image and segregates with disease phenotype under the recessive damaging model.


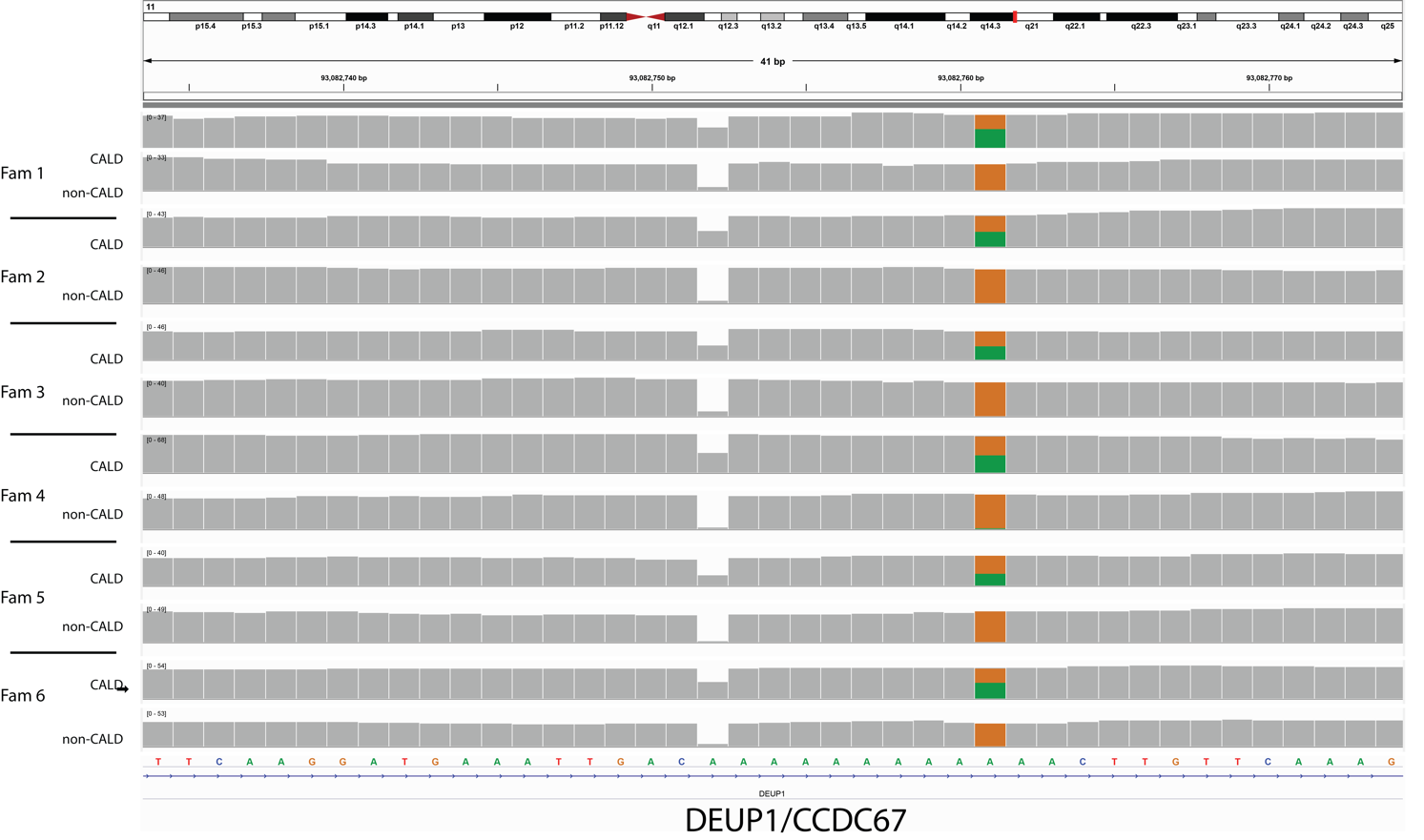


**Supplementary Figure 4.** An SNV/indel combination within *CCDC67* depicted in an IGV snapshot, showing the second discordant genotype in all families. The indel emerges as a single genotyped allele difference due to the homopolymer track from GATK. This variant is within *CCDC67* (*DEUP1*) intronic region, and segregates as a recessive protective variant. The two variants within *CCDC67* appear as an artefact of the homopolymer shortening, and are only reported as one variant from GATK HaplotypeCaller.


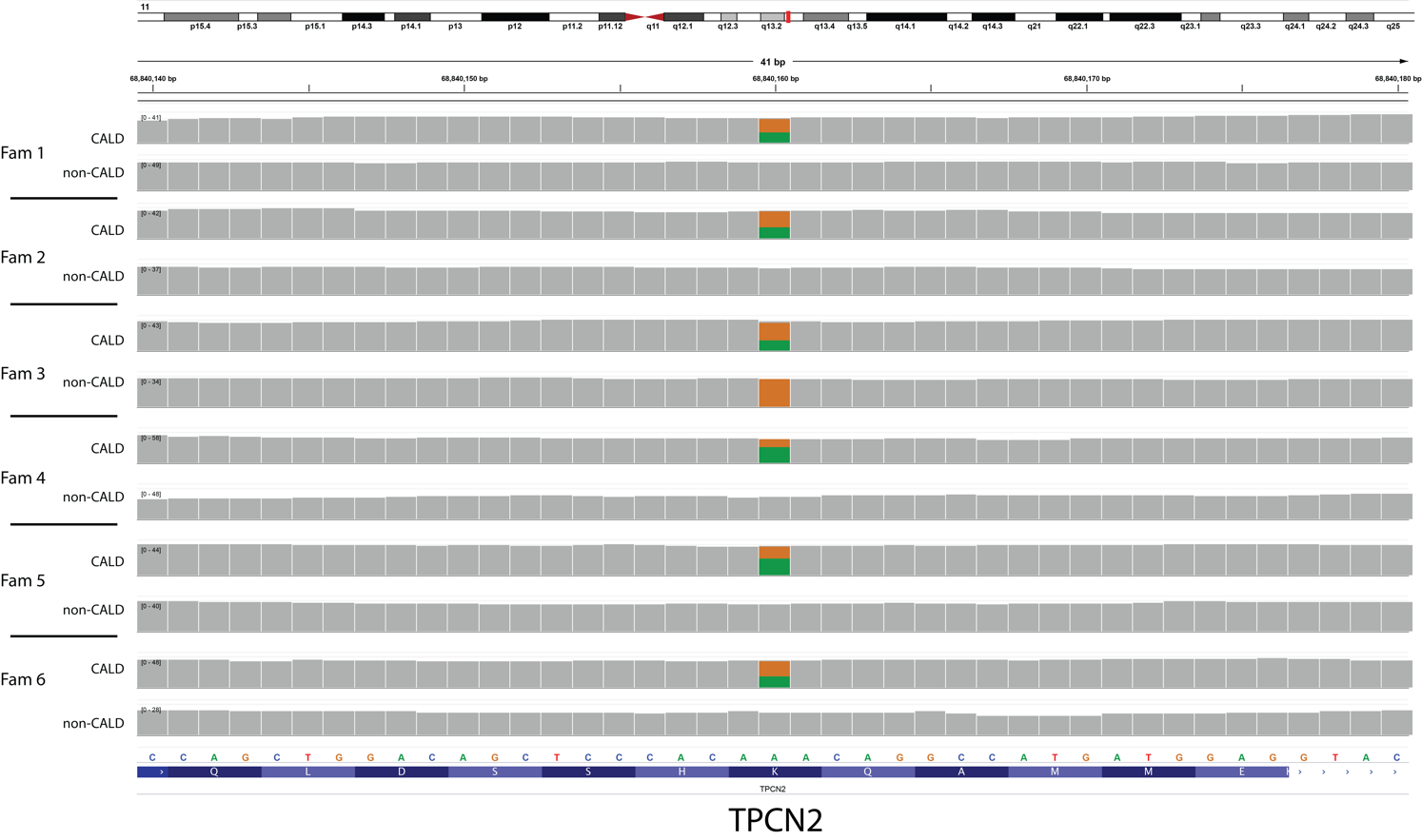


**Supplementary Figure 5.** Segregating missense variant in *TPCN2* shared by all CALD patients depicted in IGV. The only patient which carries this variant in the homozygous state is the non-CALD patient of Family 5.


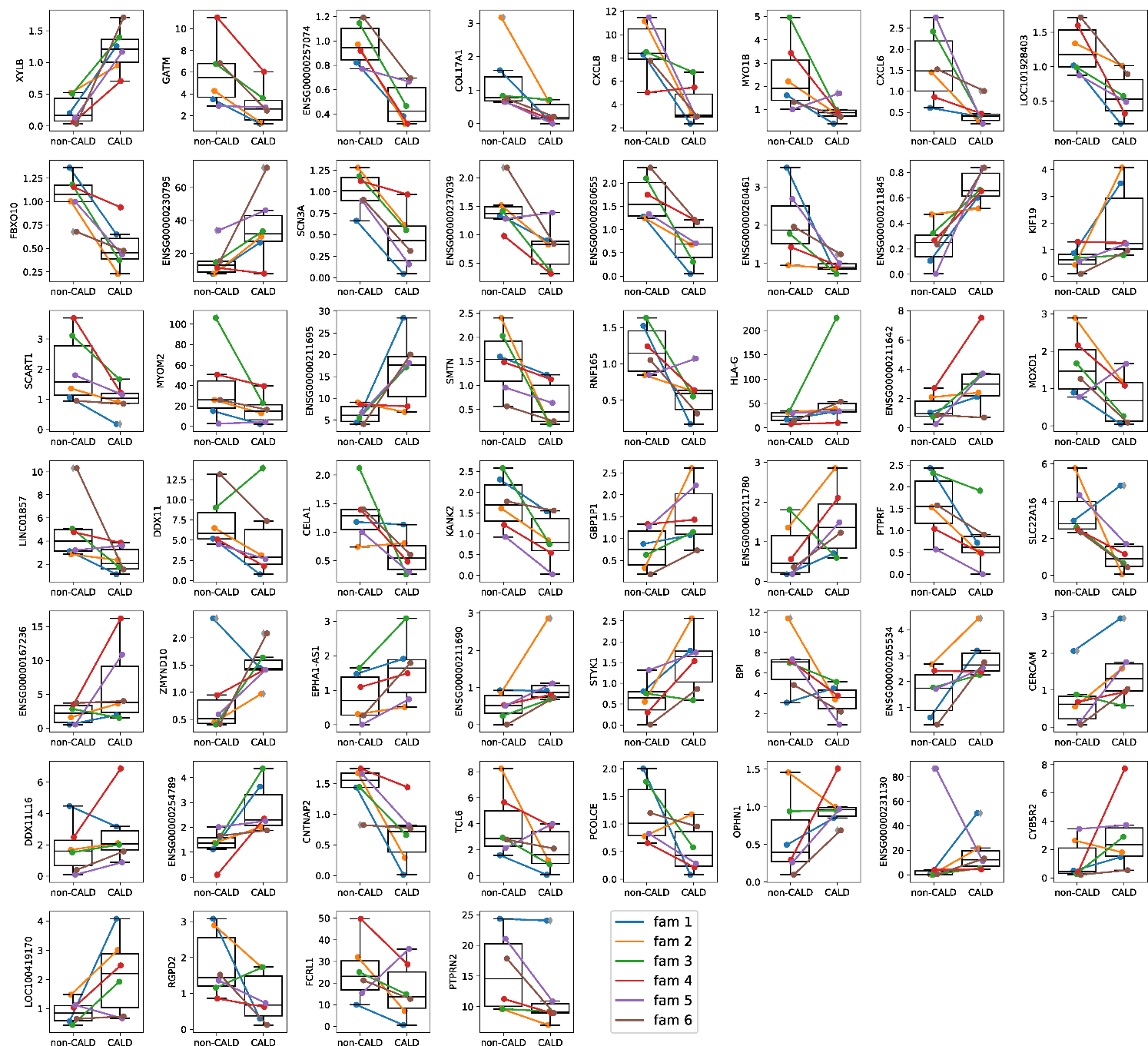


**Supplementary Figure 6.**RNA expression boxplots for the top differentially expressed genes. Each subplot contains normalized expression values for each patient in the non-CALD (left) and CALD (right) phenotype groups. Each point is coloured by family and connected by a line showing the slope of the difference between the two brothers. Value on Y-axis is transcripts per million (TPM).

**
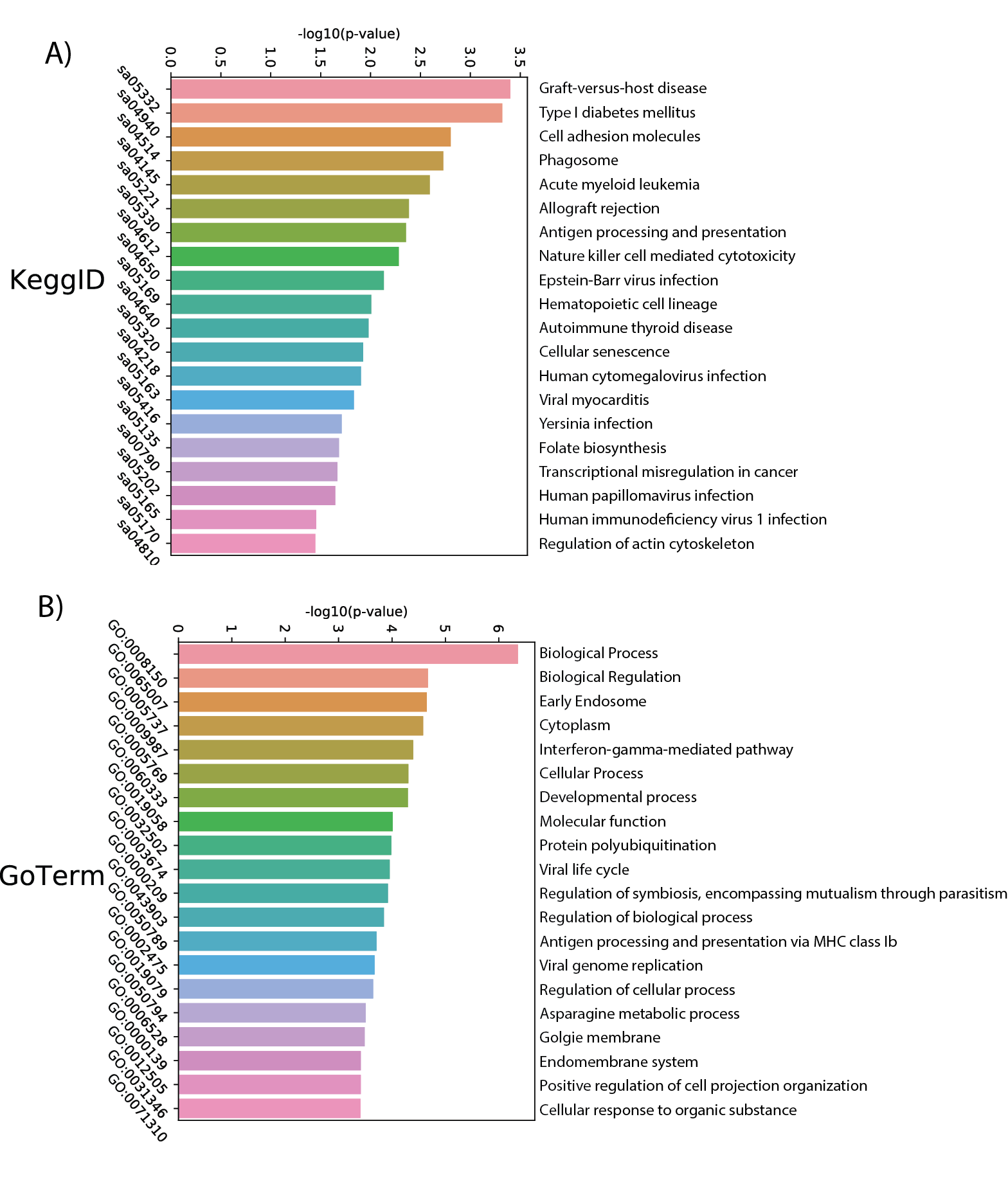
**

**Supplementary Figure 7.** KEGG (A) and GO (B) terms from RNA-seq for significant genes (p-value < 0.05) identified through differential expression of RNAseq. Plotted as bar plots showing the different terms and the -log10(p-value).

**
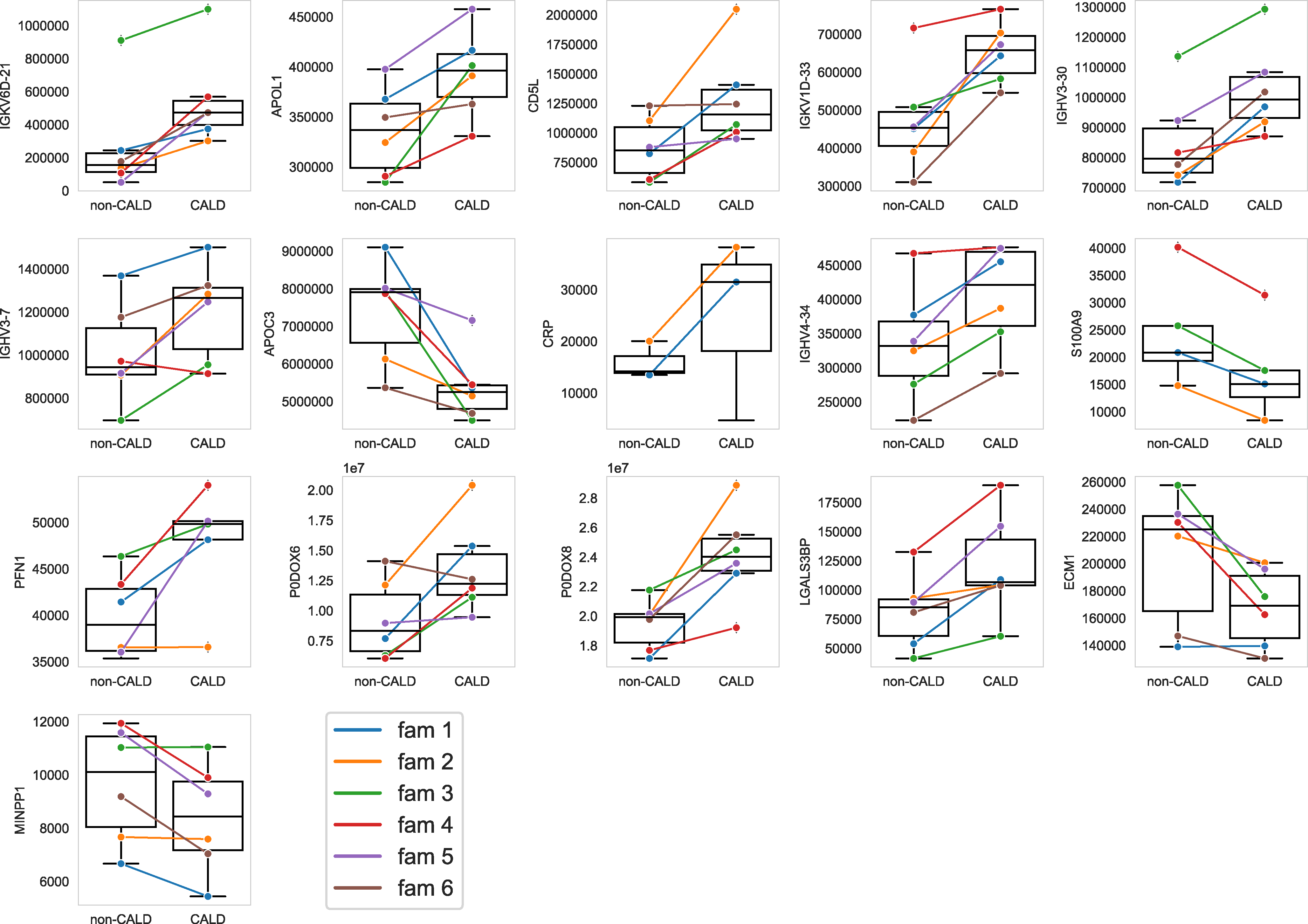
**

**Supplementary Figure 8.** Significantly different proteins shown as boxplots for all proteins which passed the threshold of p-value < 0.05 before multiple testing correction. Each plot has the annotated family values as coloured lines. Some plots have missing values for families, and if no protein was measured for both siblings in a family, then that family is not plotted. Plotted values are protein intensity.

**
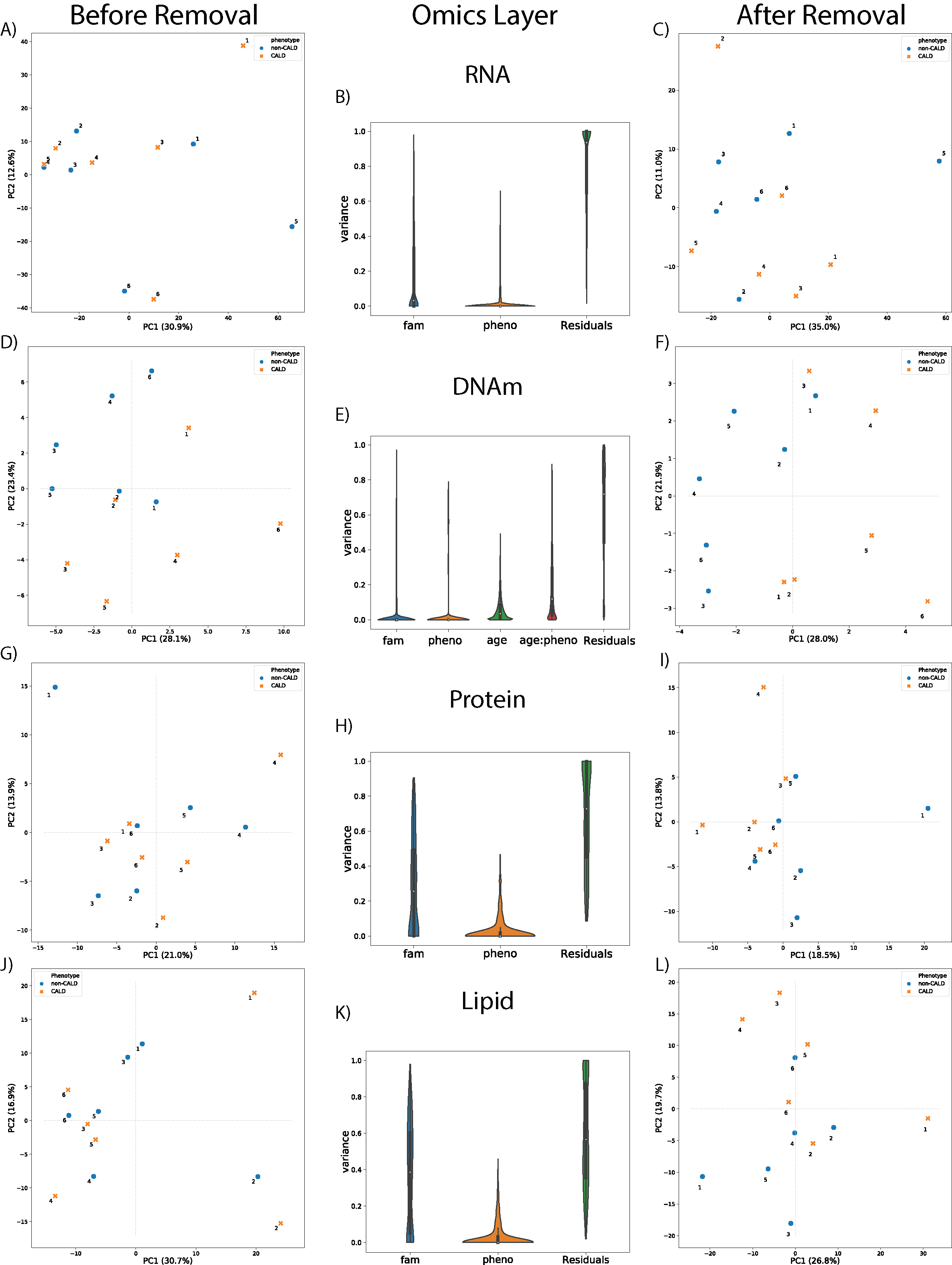
**

**Supplementary Figure 9.** Effect size calculations for transcriptomics, DNA methylation, proteomics, and lipidomics data. Principal component analysis (PCA) for each platform before (left: A, D, G, J) and after (right: C, F, I, L) removal of the family effect with non-CALD (blue) and CALD (orange) patients. For each dataset, violin plots representing the variance partitioned to the family (fam: blue), phenotype (pheno: orange), or residuals (green) (middle: B, E, H, K). DNAm has additional categories for the effect of age (green), and age-by-phenotype (red), with residuals shown in purple. After removal of the family effect, there is still no clear grouping of the data with the PCA plots which indicates a weak phenotypic effect not driven by consistent small signals across several genes. For each platform, the variance partitioned between the modeled components shows a very small contribution from the phenotype in the majority of cases.


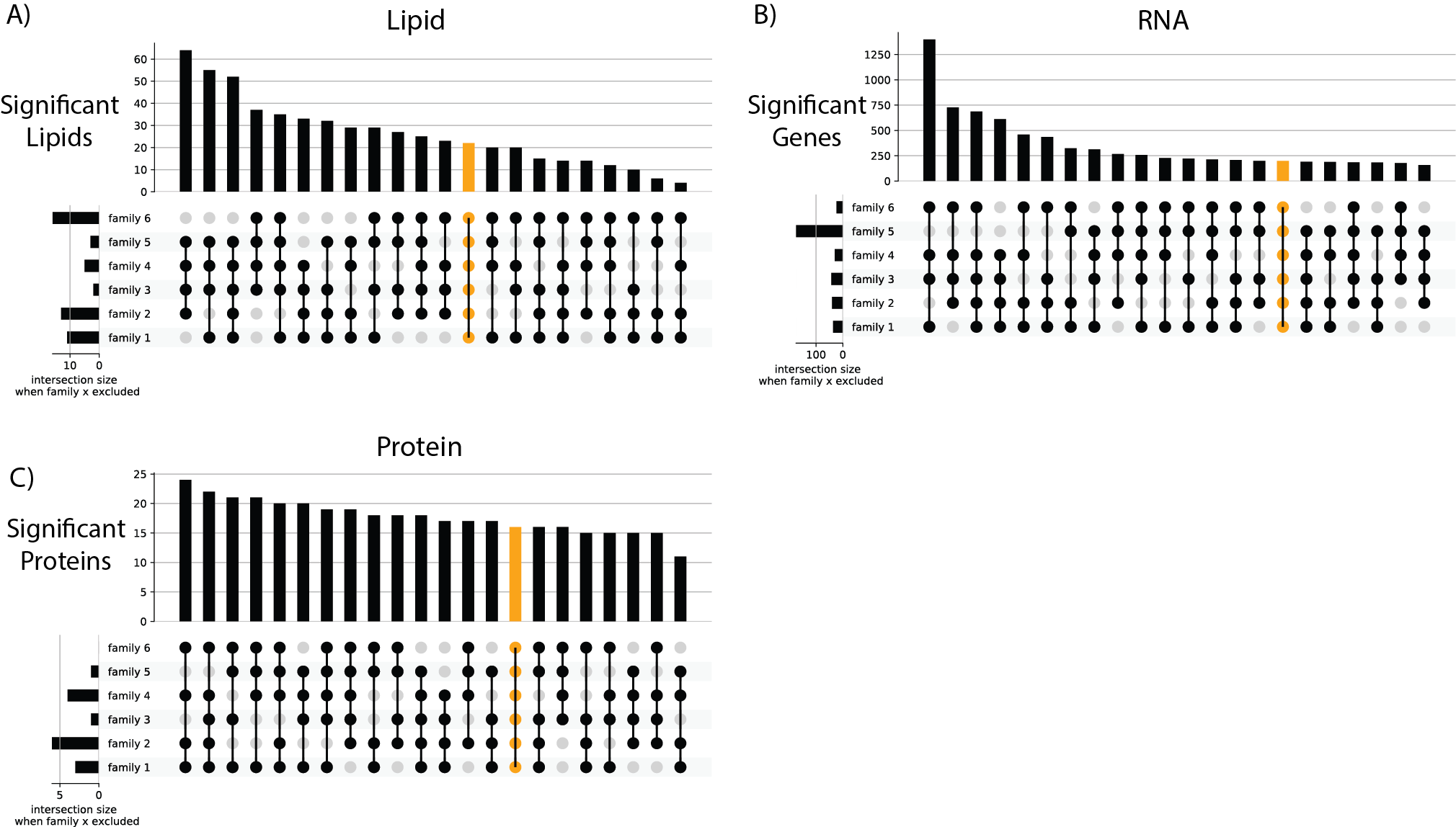


**Supplementary Figure 10.** Effect of leaving-out one or two families and repeating the univariate comparison for the lipid, RNA, and protein comparisons (B,D,F). The intersection count represents the number of lipids (B),_genes (D), and proteins (F) differentially expressed from the group of samples in the comparison (bottom connected dots). The total count shows the number of features differentially expressed in any subset comparison which a specific family is involved in.

**
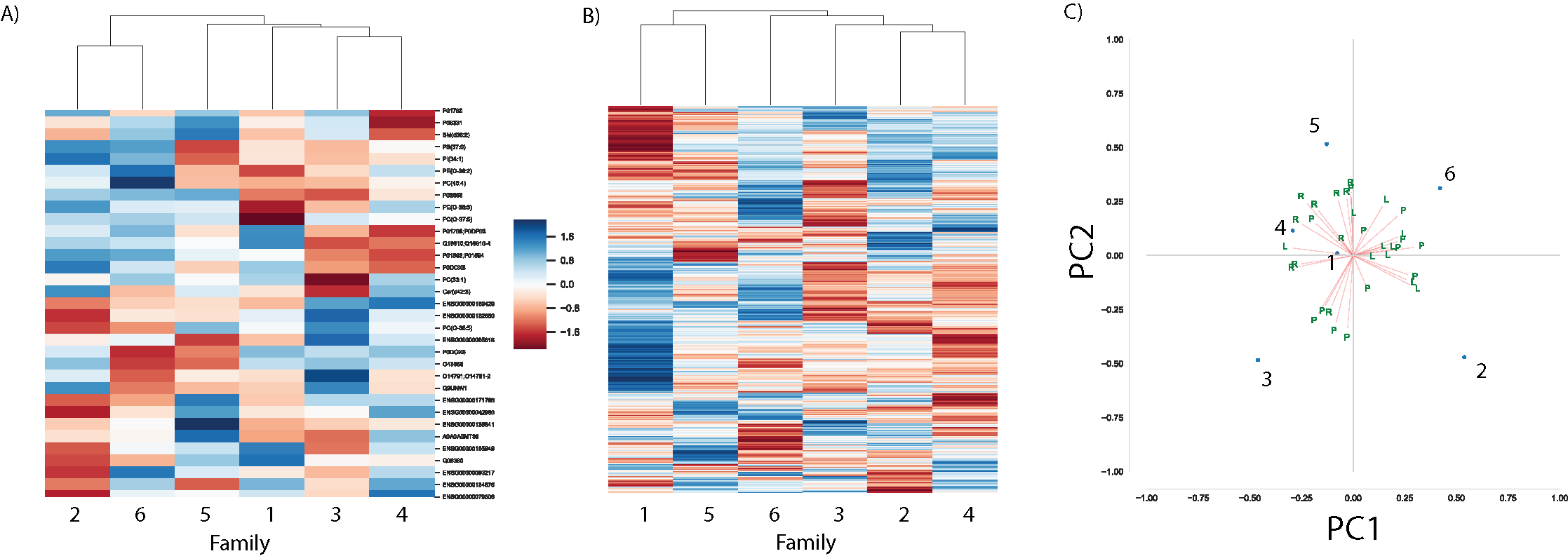
**

**Supplementary Figure 11.** Clustering top hits from intra-family differences from each platform Log2FoldChange(CALD/non-CALD) (LFC) was collected for lipid, RNA, and protein abundances for each family. These are then plotted as clustergrams with a heatmap scaling showing the LFC for each brother pair. A) the top 10 hits from the lipid, protein, and mRNA platforms. B) All significant hits with p-value < 0.05, and C) the top 10 hits as a principal component analysis (PCA) plot, with each feature (L=Lipid, P=Protein, R=RNA) labeled that contributes to the projection.

# Supplementary Tables

| lipid identifier | p-value | log2FC(ALD/CTRL) |
| --- | --- | --- |
| SM(d44:1) | 1.97E-09 | 4.95 |
| PC(46:5) | 9.29E-07 | 7.94 |
| PC(42:2) | 1.21E-06 | 6.60 |
| PC(O-43:4) | 1.63E-06 | 3.47 |
| PC(44:4) | 2.59E-06 | 4.57 |
| PC(44:3) | 2.74E-06 | 10.07 |
| PC(44:2) | 3.30E-06 | 10.96 |
| S1P(17:0) | 3.77E-06 | -0.68 |
| PC(46:4) | 5.98E-06 | 11.75 |
| LPA(18:1) | 6.39E-06 | -2.04 |

**Table S1**

Top 10 of differentially abundant lipids in ALD vs. Control. These lipids separate ALD subjects from control subjects with their p-value (adjusted). Also shown is the log2 fold change for ALD / Control, meaning values greater than 0 indicate higher abundance in ALD.

| lipid identifier | p-value | log2FC(CALD / non-CALD) |
| --- | --- | --- |
| PC(33:1) | 0.0026 | -0.457 |
| PC(O-36:5) | 0.0035 | -0.600 |
| PE(O-36:3) | 0.0076 | -0.295 |
| PE(O-36:2) | 0.0085 | -0.420 |
| PC(O-37:5) | 0.0134 | -1.131 |
| PS(37:0) | 0.0138 | -0.379 |
| PI(34:1) | 0.0142 | -0.517 |
| PC(40:4) | 0.0156 | -0.399 |
| Cer(d42:3) | 0.0228 | -0.424 |
| SM(d36:2) | 0.0239 | -0.326 |

**Table S2**

Top 10 of differentially abundant lipids between CALD and non-CALD subjects.

The p-values are uncorrected for multiple testing, sorted by significance, showing the log2 fold change of CALD over non-CALD, so negative values are lower in CALD.

**Table S3 (Excel)**
SNV and indel discordance from GATK HaplotypeCaller approach and DeepVariant approach, structural variants (SVs) called using smoove, and mobile element insertions (MEIs) called using MELT. Each category is described in Figure 3, and is displayed as a comparison between the two siblings in each family.

**(Excel)
Table S4**

Intersections of size 1-6 from the discordant variants described in Table S3, where intersect count of 1 are variants discordant and private to a single family, and size 6 are present in every family.

**(Excel)
Table S5**
Mitochondrial analysis with MToolbox.

**(Excel)**

**Table S6**
Variants shared by all CALD patients, present in a subset of non-CALD patients. Variants are annotated by being involved in an eQTL, or having a high or medium impact as annotated by GEMINI.

| ENSEMBL Identifiers | Gene | p-value | log2 FC (CALD / non-CALD) |
| --- | --- | --- | --- |
| ENSG00000093217 | *XYLB* | 0.001 | 2.545 |
| ENSG00000171766 | *GATM* | 0.001 | -1.069 |
| ENSG00000042980 | *ADAM28* | 0.004 | -0.546 |
| ENSG00000065618 | *COL17A1* | 0.005 | -2.242 |
| ENSG00000169429 | *CXCL8* | 0.005 | -1.204 |
| ENSG00000128641 | *MYO1B* | 0.006 | -1.343 |
| ENSG00000124875 | *CXCL6* | 0.006 | -1.711 |
| ENSG00000079308 | *TNS1* | 0.007 | -0.702 |
| ENSG00000132680 | *KHDC4* | 0.007 | -0.643 |
| ENSG00000165949 | *IFI27* | 0.007 | 0.993 |

**Table S7**
Top 10 differentially expressed genes from RNA-seq

The p-value is unadjusted, and the log2 fold change (LFC) is as a ratio of CALD / non-CALD, so LFC > 0 is higher in CALD, and LFC < 0 is higher in non-CALD.

| Gene | p-value | delta beta |
| --- | --- | --- |
| *RGS14* | 8.63E-08 | 0.258 |
| *PTPRN2* | 4.93E-08 | 0.232 |
| *HLA-DQB1* | 1.13E-03 | -0.231 |
| *HCG4P6* | 5.82E-17 | -0.176 |
| *LOC100507140* | 4.96E-09 | -0.175 |
| *MSH5* | 4.18E-05 | -0.159 |
| *PRHOXNB* | 1.21E-49 | 0.155 |
| *LOC100507506* | 1.25E-07 | -0.153 |
| *JPH3* | 1.87E-05 | -0.153 |
| *TCERG1L* | 3.80E-04 | 0.151 |

**Table S8**
Top 10 differentially methylated DMRs mapped to named genes, positive delta beta means CALD is higher than non-CALD. The DMRs with the largest delta betas are being shown here.

| Gene | Protein Accessions | p-value | log2 FC (CALD / non-CALD) |
| --- | --- | --- | --- |
| *IGKV6D-21* | A0A0A0MT36 | 0.005 | 1.517 |
| *.* | P0DOX6 | 0.046 | 0.577 |
| *CD5L* | O43866 | 0.031 | 0.568 |
| *LGALS3BP* | Q08380 | 0.010 | 0.566 |
| *IGKV1D-33;IGKV1-33* | P01593;P01594 | 0.007 | 0.506 |
| *.* | P0DOX8 | 0.007 | 0.304 |
| *IGHV4-34* | P06331 | 0.008 | 0.297 |
| *IGHV3-30;IGHV3-30-5* | P01768;P0DP03 | 0.002 | 0.274 |
| *IGHV3-7* | P01780 | 0.028 | 0.270 |
| *PFN1(1)* | P07737 | 0.049 | 0.256 |
| *APOL1* | O14791;O14791-2 | 0.009 | 0.231 |
| *CRP(6)* | P02741 | 0.003 | 0.187 |
| *MINPP1* | Q9UNW1 | 0.029 | -0.213 |
| *ECM1* | Q16610;Q16610-4 | 0.035 | -0.270 |
| *APOC3* | P02656 | 0.015 | -0.454 |
| *S100A9(2)* | P06702 | 0.002 | -0.560 |

**Table S9**Proteomic hits, the 16 proteins with differential abundance (p<0.05), positive fold changes indicate up-regulated for CALD. The parentheses indicate the number of samples without a value.

**(Excel)
Table S10**Viral Analysis with Centrifuge for each of the patients in the study. Separated by family, and split left/right for CALD/non-CALD respectively. Top hits identified from centrifuge are shown.

# Supplementary Results

## Potential physiological roles for candidate alleles

Through the allele discordance analysis we identified two genes (*WIBG* and *DEUP1*) with low-impact variants that segregate in all families. The variant within *DEUP1* is a deep intronic variant (not splice-region collocalized), located within a homopolymer stretch. Nearby genes in the region include *SLC36A4* and *SMCO4*. The variant within *WIBG* is within the downstream intergenic region of the gene, without predicted functional impact (CADD score 0.623) (Kircher, Witten et al. 2014). Nearby genes in the region include *DGKA*, *PMEL*, *CDK2*, *TMEM198B*, and *ORMDL2*. Neither variant is associated with eQTLs from the GTEx catalog (Gamazon, Segrè et al. 2018), lessening the chance that these variants impact the surrounding genes. Furthermore, neither variant is mentioned within the GWAS catalog (Buniello, MacArthur et al. 2019). In the shared CALD allele analysis we found one gene (*TPCN2*) which has an impactful variant and is shared by all CALD patients in the heterozygous state, and absent in all non-CALD families except family 4. Based upon literature examination, none of these genes have been associated with ALD previously. *TPCN2* colocalizes to lysosomal membranes and plays a role in calcium ion release from lysosomes, so it’s role within adrenoleukodystrophy--a lysosomal storage disorder--could be promising.

# Supplementary Methods

## Sample Collection

Sample material for WGS, EPIC and RNA was extracted from lymphocyte pellets (drawn from whole blood) while sample material for PROT and LIP was taken from plasma that was extracted at the same time as the lymphocyte pellets. Measurements on each different platform were performed by different laboratories. The WGS and RNA sequences were measured at Macrogen. The EPIC data was measured at the Kobor Lab UBC. The lipidomics data was measured and processed at the lab GMZ, AMC. Control samples for lipidomics were taken from healthy adult male donors through the Amsterdam UMC Hospital and profiled alongside the ALD samples within this study. Proteomic data was generated at the Department of Pathology and Laboratory Medicine at UBC.

## RNA-seq analysis

RNA extraction was performed on lymphocytes pellets (drawn from whole blood) of the 12 subjects. The raw data statistics indicate an overall good quality of the data. The sequence data were mapped against the hg19 reference genome using the STAR 2-pass algorithm to align spliced reads with short overhangs across previously detected junctions (Dobin, Davis et al. 2013). To count the sequences with featureCounts (Liao, Smyth et al. 2014) GRCh37.p13 (Homo_sapiens.GRCh37.87.gtf) was used to define coding and non-coding sequences of the genes (57,905 in total). From the 57,905 unique definitions, 23,136 were not found in any sample. Further gene selection was achieved by limiting to those genes that were found in one or both groups with at least 0.5 cpm per sample. This way 12,988 sequences remained for further analysis. The sequences were tested for differential expression using the R-package edgeR (Robinson, McCarthy et al. 2010, Liao, Smyth et al. 2014). The MDS plot from the limma package (Ritchie, Phipson et al. 2015) validated the strong family effect (grouping), which indicates the necessity for including this factor in the regression model. The expression of the sequences (assigned with an Ensembl code ENSGXXX) were modeled using equation 2.

### Enrichment Analysis

Enrichment analysis in GO and KEGG terms for the up and down regulated genes (with p-value <0.05, no multiple testing correction) in the CALD group with respect to the non-CALD group was performed using the functions goana, kegga from the limma package (Ritchie, Phipson et al. 2015) in R.

### Viral Analysis

Viruses and bacteria expressing at a detectable level in RNA-seq reads were identified using Centrifuge (v1.0.3) (Kim, Song et al. 2016) using the default viral + bacterial + human database. Differences in abundance were qualitatively examined between brother pairs.

## Methylation analysis

Illumina MethylationEPIC array (850k) analysis was performed on the DNA samples extracted from purified lymphocytes (whole blood) of the 12 subjects. The 12 bisulfite-converted samples were randomly distributed across 2 beadchips and all processed in 1 batch. Following raw data import into GenomeStudio (Illumina, San Diego, CA), signals were color-corrected and background subtracted using the built-in array control probes, and subsequently imported into *RStudio* in the form of an average β-value matrix, with each value representing the percent methylation at a given CpG measured in the corresponding sample. 
 Further data preprocessing was performed in *RStudio* 3.5.2, where SNP clustering was first used to confirm the siblings relatedness using the 59 rs probes present on the EPIC arrays. Then, we filtered out SNP and XY probes due to their differences in beta-value distributions, and also removed cross-hybridizing and polymorphic probes to facilitate the downstream normalization steps (Pidsley, Zotenko et al. 2016). Additionally, poorly-performing probes were identified and removed using the *pfilter* function (*wateRmelon package*) using the default settings. 786,197 probes were carried forward for intrasample (quantile) and intersample (Beta-Mixture Quantile; BMIQ) (Teschendorff, Marabita et al. 2013) normalizations to reduce technical variations and probe-type bias, and batch-corrections using *ComBat* (Johnson, Li et al. 2007).  
 The purified lymphocyte samples consist of several classes of agranulocytes, including T cells (CD4+ Helpers, CD8+ cytotoxic), B cells, monocytes, and natural killer cells. These lineages differ significantly in their DNA methylation profiles and the mixture of them, at unknown concentrations, may confound DNA methylation analyse, especially since differences in immunity may cause these cell proportions to fluctuate. To correct for differences in these blood cell types, we performed bioinformatics-based blood cell prediction based on the DNAm profile of these samples (Houseman, Accomando et al. 2012), and subsequently adjusted the beta-values in each sample using a regression-based method as described in (Jones, Islam et al. 2017). To assess differentially methylated regions (DMRs) between individuals at different disease progressions, the package *DMRcate* (Peters, Buckley et al. 2015) was used with following linear regression model: (*M-value* ~ cALD Status + Age + Family), where *M-value* represent logit-transformed beta-values at each CpG after cell-type corrections (Du, Zhang et al. 2010). The observed DMRs are identified with an FDR cutoff of <0.05 (Benjamini-Hochberg), and aggregate delta beta >10%.

## Lipidomics Analysis

The plasma samples from the 12 ALD patients and 8 control subjects were measured on a lipidomic platform in three different modes (normal phase, positive and negative mode, reversed phase positive mode) and processed using an XCMS (Smith, Want et al. 2006) based in-house automated pipeline (Huffnagel, Dijkgraaf et al. 2019). The peaks were annotated and putatively identified based on retention time and exact mass. Combining the results of the three different modes resulted in thousands of peaks (peak-groups) from which 1160 were (putatively) identified. Further cleaning consisted of removing lipids that were below the detection limit detected in some of the ALD samples. Lipids that were only detected in one of the two groups however, were included. 546 lipids remained for further analysis.

## Proteomics Analysis

### Sample preparation for mass spectrometry analysis

1 µl from each sample was combined and depleted of abundant plasma proteins using the Top 2 Abundant Protein Depletion Spin Columns (Pierce TM) according to the manufacturer’s protocol. 50 µl of depleted sample pool was lysed in SDS buffer (50 mM HEPES pH 8.5, 2% SDS< 1X protease inhibitor) and protein extraction and digestion was performed as follows: heat at 95oC for 5 min, incubate in benzonase for 30 min at 37oC, incubate in 10 mM DTT for 30 min at 37 oC, alkylate with 50 mM CAA for 30 min at room temperature in the dark. Protein binding and digestion using Single-pot solid-phase-enhanced sample preparation (SP3) magnetic beads was performed as described elsewhere (Weng, Demir et al. 2019). Briefly, 1:1 v/v ratio of hydrophilic (conc. 10μg/μL, GE Life Sciences, cat. no. 4515-2105-050250) and hydrophobic Sera-Mag SpeedBeads carboxylate-modified magnetic beads (conc. 10μg/μL, GE Life Sciences, cat. no. 6515-2105-050250) were prepared, bound to proteins in 80% ethanol, washed, and incubated overnight in 20 mM HEPES (pH 8.5) and trypsin (Promega, cat. no. V5113) at 37 oC.

For individual samples, 38 µl of SDS lysis buffer was added to 2 µl of each undepleted sample, followed by heating, reduction, alkylation, on-bead SP3 protein binding, and trypsin digestion, as described earlier.

C18 sample clean-up was performed on all acidified samples (pH 3-4) using Nest Group spin columns (Nest Group Inc, cat. no. HEM S18V), eluted in 60% ACN, 0.1FA, and dried in a speed vacuum. Samples were suspended in 0.1% FA in water (Thermo Scientific, cat. no. SC2352911) and spiked with Biognosys iRT peptides prior to data independent mass spectrometry analysis on 0.8 µg of sample peptides.

### High pH Fractionation

Sample pool was fractionated using a Kinetic EVO C18 column (2.1mm×150mm, 1.7μm core shell, 100Å pore size, Phenomenex) connected to an Agilent 1100 HPLC system equipped with a diode array detector (254, 260, and 280 nm). Fractionation was done at a flow rate of 0.2ml per minute using a gradient of mobile phase A (10mM ammonium bicarbonate, pH 8, Fisher Scientific, cat. no. BP2413-500) and mobile phase B (acetonitrile, Sigma-Aldrich, cat. no. 34998-4L) from 3% to 35% B over 60 min. The 48 fractions collected per minute across the elution window were concatenated to a set of 12 new fractions (e.g. fractions 1+13+25+37 as new fraction 1). The 12 fractions were acidified and cleaned using stage-tips. To prepare stage-tips, four small circular EmporeTM SPE C18 disks (Sigma, Cat. No. 66883-U) were punched with a flat-end Hamilton needle (Cat. No. 90517), and a straightened paper clip was used to gently push down the C18 disks into a VWR P200 pipette tip (Cat. No. 89079-474). Following elution in 60% acetonitrile, 0.1% FA, samples were dried in a speed vacuum. Prior to data dependent mass spectrometry analysis, dried samples were reconstituted in 0.1% FA in water and spiked with iRT peptides. 0.2 µg of sample peptides from each fraction was analyzed.

### Mass Spectrometry Analysis

Mass spectrometry analysis was performed on an Orbitrap Lumos platform (Thermo Scientific) coupled to an Easy-nLC 1200 system (Thermo) and EasySpray source (Thermo). Sample separation was done using a 50 µm ID x 50 cm EasyNano column (Thermo) at 50 oC.

Prior to each sample injection, the analytical column was equilibrated at 750 bar for a total volume of 6 μL. Inject volumes were 4µl (~800ng) with a total loading volume of 10μL at a pressure of 750 bar. Peptide separation was performed on gradient of mobile phase A (water and 0.1% formic acid) and mobile phase B (0.1% formic acid in 80% acetonitrile) at a flow rate of 0.3 µl/min. For DDA, mobile phase B setting was as follows: 1-7%, 0-2min; 7-21%, 2-97min; 21-34%, 97-117min; 34-100%, 117-121min; 100%, 121-133min; 100-5%, 133-134min. Gradient of mobile phase B for DIA was: 5-7%, 0-10min; 7-21%, 10-135min; 21-34%, 135-170min; 34-100%, 170-180min; 100%, 180-192min; 100-5%, 192-194min.

The Lumos (control software version 3.1.2412.17) was operated with a positive ion spray voltage of 2400 and a transfer tube temperature of 325°C.

For DDA acquisition, full-scan MS spectra (MS1) were acquired in the Orbitrap at a resolution of 60K across a mass range of 400 – 1800 m/z, RF lens setting of 30, an AGC target of 1e6, max injection time of 75ms in profile mode with IC on, and default charge state was set to 2. For MS2 scans, charge state filtering of 2 – 6 and dynamic exclusion for 20 seconds with 10ppm tolerances was used with a 1.4 m/z window prior to HCD fragmentation of 28%.  MS2 data acquisition carried out in the Orbitrap used a 15K resolution, scan range 200-2000m/z, an AGC target of 5e4, and a max injection time of 50ms in profile mode with parallelizable time turned off.

For DIA acquisition, a survey scan followed by 3 DIA scans was used.  Survey scans (MS1) were acquired in the Orbitrap at a resolution of 120K across a mass range of 300 – 1650 m/z, RF lens setting of 60, an AGC target of 1e6, max injection time of 50ms in profile mode with IC on. For DIA scan 1, precursor mass range was 400-800 and quadrupole isolation window 22m/z, mass defect 1 with HCD fragmentation, collision energy 30% was used.  OT resolution was set at 15000 with RF lens 60%, AGC target 5e4, with maximum inject time 60ms and scan range 200-2000 in profile mode, loop control set to all and IC off. DIA scan 2 was similar but with precursor mass range 800-1080m/z and isolation window 50m/z. Likewise with DIA scan 3 with precursor mass range 1080-1380m/z and isolation window 100m/z.

### Processing of mass spectrometry data.

Raw DDA and DIA data were processed and searched with Spectronaut Pulsar (version X, Biognosys, Schlieren, Switzerland). A spectral library was first generated by searching all DDA and DIA raw files in Spectronaut Pulsar with the default settings: digest type = Trypsin/P, minimum peptide length = 7, missed cleavages = 2, modifications = Carbamidomethyl (C) as fixed, and Acetyl (Protein N-term), Oxidation (M) as variable. The resulting spectral library containing precursor and fragment details and normalized retention times, was used for targeted analysis of DIA data acquired on individual samples. Spectronaut default DIA settings were applied. Briefly, this consisted of a “dynamic” setting with correction factor of 1 for MS1 and MS2 tolerance strategy, and for extracted ion chromatogram retention time window. Precision iRT was activated for calibration of MS run with local (non-linear) regression. Feature identification was done using the ‘mutated’ decoy method, with ‘dynamic’ strategy and library size fraction of 0.1. False discovery rate for precursors and proteins were 1% respectively. Protein quantification reports generated from Spectronaut were used for further statistical analysis.

### Proteomics Quality Analysis

Plasma sample preparation for mass spectrometry analysis were prepared according to standard protocol. High performance liquid chromatography (HPLC) and mass spectrometry (MS) analytical set-up were maintained at optimal conditions, as confirmed using quality control standards (PierceTM HeLa protein Digest Standard). Peptide solutions from each plasma sample were spiked with index reference time peptides (iRT peptides, Biognosys, Switzerlend). iRT peptides were used to monitor retention time precision and quantitative resolution of eluted peptides. iRT peptides were also used to confirm gradient stability and quantitative reproducibility of protein data across samples.

## DNA sequencing analysis

### Read mapping and tailoring

WGS reads were mapped against the hg19 reference genome using BWA mem (v0.7.12) (Li and Durbin 2009), converted to binary format and sorted using samtools (v1.3.1,) (Li, Handsaker et al. 2009), duplicate marked using PicardTools (v2.18.13) (<http://broadinstitute.github.io/picard/>), and realigned using GATK (3.4.3) (Poplin, Ruano-Rubio et al.).

### Variant Calling & Interpretation: SNV/Indel

The mapped reads were jointly genotyped using GATK HaplotypeCaller --GVCF and GenotypeGVCFs (v3.4.3) (Poplin, Ruano-Rubio et al.) to produce a set of SNVs and small indels. The resulting merged VCF was split and normalized using VT (v0.5), and annotated using SNPeff (v4.3t, gene annotation GRCh37.87) (Cingolani, Platts et al. 2012)and VCFAnno (v0.2.8) (Pedersen, Layer et al. 2016) against a set of custom databases (for more information see (van Kuilenburg, Tarailo-Graovac et al. 2019)). The merged, normalized, annotated variant file was then converted into a GEMINI database (v0.19.1) (Paila, Chapman et al. 2013)using VCF2DB (v0.0.1). Intra-family variant comparisons (between brothers) were performed using GEMINI, and then intersected across families using InterVene (v0.6.4) (Khan and Mathelier 2017). The mapped reads were also genotyped independently using DeepVariant (v0.8.0) (Poplin, Chang et al. 2018), and merged using BCFTools with the -0 flag. Annotation and genotype filtering were then repeated as described above.

### Structural variants (SVs)

SVs were called from the mapped read files using smoove (v0.2.3, <https://github.com/brentp/smoove>).  SVs were annotated for genomic effect using AnnotSV (v2.1) (Geoffroy, Herenger et al. 2018). Comparisons between individual samples were run using custom scripts based upon the merged and annotated SV table.

### Mobile element insertions (MEIs)

Mobile element insertions were called from the mapped read files using MELT (v2.5.1) (Gardner, Lam et al. 2017), searching for Alu, LINE and SINE insertions. The same scripts from the SV calling section were used for comparisons between individuals from the annotated MEI table.

### Mitochondrial analysis

Mitochondrial analysis was performed on the unmapped reads using MToolbox (v1.0.0) (Picardi and Pesole 2012).

### Whole Genome Sequencing Quality Control Metrics

Quality metrics were gathered at the variant calling (SNV/Indel) level for each of the 12 samples, 6 brother pairs, sequenced within this study. Metrics include ancestry inference, sex determination, and relatedness between individuals. These metrics ensured that sample labeling was correct, and variant calls were of sufficient quality.

# Supplementary References

Buniello, A., J. A. L. MacArthur, M. Cerezo, L. W. Harris, J. Hayhurst, C. Malangone, A. McMahon, J. Morales, E. Mountjoy, E. Sollis, D. Suveges, O. Vrousgou, P. L. Whetzel, R. Amode, J. A. Guillen, H. S. Riat, S. J. Trevanion, P. Hall, H. Junkins, P. Flicek, T. Burdett, L. A. Hindorff, F. Cunningham and H. Parkinson (2019). "The NHGRI-EBI GWAS Catalog of published genome-wide association studies, targeted arrays and summary statistics 2019." Nucleic Acids Res. **47**(D1): D1005-D1012.

Cingolani, P., A. Platts, L. L. Wang, M. Coon, T. Nguyen, L. Wang, S. J. Land, X. Lu and D. M. Ruden (2012). "A program for annotating and predicting the effects of single nucleotide polymorphisms, SnpEff: SNPs in the genome of Drosophila melanogaster strain w1118; iso-2; iso-3." Fly **6**(2): 80-92.

Dobin, A., C. A. Davis, F. Schlesinger, J. Drenkow, C. Zaleski, S. Jha, P. Batut, M. Chaisson and T. R. Gingeras (2013). "STAR: ultrafast universal RNA-seq aligner." Bioinformatics **29**(1): 15-21.

Du, P., X. Zhang, C.-C. Huang, N. Jafari, W. A. Kibbe, L. Hou and S. M. Lin (2010). "Comparison of Beta-value and M-value methods for quantifying methylation levels by microarray analysis." BMC Bioinformatics **11**: 587.

Gamazon, E. R., A. V. Segrè, M. van de Bunt, X. Wen, H. S. Xi, F. Hormozdiari, H. Ongen, A. Konkashbaev, E. M. Derks, F. Aguet, J. Quan, G. T. Consortium, D. L. Nicolae, E. Eskin, M. Kellis, G. Getz, M. I. McCarthy, E. T. Dermitzakis, N. J. Cox and K. G. Ardlie (2018). "Using an atlas of gene regulation across 44 human tissues to inform complex disease- and trait-associated variation." Nat. Genet. **50**(7): 956-967.

Gardner, E. J., V. K. Lam, D. N. Harris, N. T. Chuang, E. C. Scott, W. S. Pittard, R. E. Mills, C. Genomes Project and S. E. Devine (2017). "The Mobile Element Locator Tool (MELT): population-scale mobile element discovery and biology." Genome Res. **27**(11): 1916-1929.

Geoffroy, V., Y. Herenger, A. Kress, C. Stoetzel, A. Piton, H. Dollfus and J. Muller (2018). "AnnotSV: an integrated tool for structural variations annotation." Bioinformatics **34**(20): 3572-3574.

Houseman, E. A., W. P. Accomando, D. C. Koestler, B. C. Christensen, C. J. Marsit, H. H. Nelson, J. K. Wiencke and K. T. Kelsey (2012). "DNA methylation arrays as surrogate measures of cell mixture distribution." BMC Bioinformatics **13**: 86.

Huffnagel, I. C., M. G. W. Dijkgraaf, G. E. Janssens, M. van Weeghel, B. M. van Geel, B. T. Poll-The, S. Kemp and M. Engelen (2019). "Disease progression in women with X-linked adrenoleukodystrophy is slow." Orphanet J. Rare Dis. **14**(1): 30.

Johnson, W. E., C. Li and A. Rabinovic (2007). "Adjusting batch effects in microarray expression data using empirical Bayes methods." Biostatistics **8**(1): 118-127.

Jones, M. J., S. A. Islam, R. D. Edgar and M. S. Kobor (2017). "Adjusting for Cell Type Composition in DNA Methylation Data Using a Regression-Based Approach." Methods Mol. Biol. **1589**: 99-106.

Khan, A. and A. Mathelier (2017). "Intervene: a tool for intersection and visualization of multiple gene or genomic region sets." BMC Bioinformatics **18**(1).

Kim, D., L. Song, F. P. Breitwieser and S. L. Salzberg (2016). "Centrifuge: rapid and sensitive classification of metagenomic sequences." Genome Res. **26**(12): 1721-1729.

Kircher, M., D. M. Witten, P. Jain, B. J. O'Roak, G. M. Cooper and J. Shendure (2014). "A general framework for estimating the relative pathogenicity of human genetic variants." Nat. Genet. **46**(3): 310-315.

Li, H. and R. Durbin (2009). "Fast and accurate short read alignment with Burrows-Wheeler transform." Bioinformatics **25**(14): 1754-1760.

Li, H., B. Handsaker, A. Wysoker, T. Fennell, J. Ruan, N. Homer, G. Marth, G. Abecasis, R. Durbin and S. Genome Project Data Processing (2009). "The Sequence Alignment/Map format and SAMtools." Bioinformatics **25**(16): 2078-2079.

Liao, Y., G. K. Smyth and W. Shi (2014). "featureCounts: an efficient general purpose program for assigning sequence reads to genomic features." Bioinformatics **30**(7): 923-930.

Paila, U., B. A. Chapman, R. Kirchner and A. R. Quinlan (2013). "GEMINI: integrative exploration of genetic variation and genome annotations." PLoS Comput. Biol. **9**(7): e1003153.

Pedersen, B. S., R. M. Layer and A. R. Quinlan (2016). "Vcfanno: fast, flexible annotation of genetic variants." Genome Biol. **17**(1): 118.

Peters, T. J., M. J. Buckley, A. L. Statham, R. Pidsley, K. Samaras, R. V Lord, S. J. Clark and P. L. Molloy (2015). "De novo identification of differentially methylated regions in the human genome." Epigenetics Chromatin **8**: 6.

Picardi, E. and G. Pesole (2012). "Mitochondrial genomes gleaned from human whole-exome sequencing." Nat. Methods **9**(6): 523-524.

Pidsley, R., E. Zotenko, T. J. Peters, M. G. Lawrence, G. P. Risbridger, P. Molloy, S. Van Djik, B. Muhlhausler, C. Stirzaker and S. J. Clark (2016). "Critical evaluation of the Illumina MethylationEPIC BeadChip microarray for whole-genome DNA methylation profiling." Genome Biol. **17**(1): 208.

Poplin, R., P.-C. Chang, D. Alexander, S. Schwartz, T. Colthurst, A. Ku, D. Newburger, J. Dijamco, N. Nguyen, P. T. Afshar, S. S. Gross, L. Dorfman, C. Y. McLean and M. A. DePristo (2018). "A universal SNP and small-indel variant caller using deep neural networks." Nat. Biotechnol. **36**(10): 983-987.

Poplin, R., V. Ruano-Rubio, M. A. DePristo, T. J. Fennell, M. O. Carneiro, G. A. Van der Auwera, D. E. Kling, L. D. Gauthier, A. Levy-Moonshine, D. Roazen, K. Shakir, J. Thibault, S. Chandran, C. Whelan, M. Lek, S. Gabriel, M. J. Daly, B. Neale, D. G. MacArthur and E. Banks "Scaling accurate genetic variant discovery to tens of thousands of samples."

Ritchie, M. E., B. Phipson, D. Wu, Y. Hu, C. W. Law, W. Shi and G. K. Smyth (2015). "limma powers differential expression analyses for RNA-sequencing and microarray studies." Nucleic Acids Res. **43**(7): e47.

Robinson, M. D., D. J. McCarthy and G. K. Smyth (2010). "edgeR: a Bioconductor package for differential expression analysis of digital gene expression data." Bioinformatics **26**(1): 139-140.

Smith, C. A., E. J. Want, G. O'Maille, R. Abagyan and G. Siuzdak (2006). "XCMS: processing mass spectrometry data for metabolite profiling using nonlinear peak alignment, matching, and identification." Anal. Chem. **78**(3): 779-787.

Teschendorff, A. E., F. Marabita, M. Lechner, T. Bartlett, J. Tegner, D. Gomez-Cabrero and S. Beck (2013). "A beta-mixture quantile normalization method for correcting probe design bias in Illumina Infinium 450 k DNA methylation data." Bioinformatics **29**(2): 189-196.

van Kuilenburg, A. B. P., M. Tarailo-Graovac, P. A. Richmond, B. I. Drögemöller, M. A. Pouladi, R. Leen, K. Brand-Arzamendi, D. Dobritzsch, E. Dolzhenko, M. A. Eberle, B. Hayward, M. J. Jones, F. Karbassi, M. S. Kobor, J. Koster, D. Kumari, M. Li, J. MacIsaac, C. McDonald, J. Meijer, C. Nguyen, I.-S. Rajan-Babu, S. W. Scherer, B. Sim, B. Trost, L. A. Tseng, M. Turkenburg, J. J. F. A. van Vugt, J. H. Veldink, J. S. Walia, Y. Wang, M. van Weeghel, G. E. B. Wright, X. Xu, R. K. C. Yuen, J. Zhang, C. J. Ross, W. W. Wasserman, M. T. Geraghty, S. Santra, R. J. A. Wanders, X.-Y. Wen, H. R. Waterham, K. Usdin and C. D. M. van Karnebeek (2019). "Glutaminase Deficiency Caused by Short Tandem Repeat Expansion in." N. Engl. J. Med. **380**(15): 1433-1441.

Weng, S. S. H., F. Demir, E. K. Ergin, S. Dirnberger, A. Uzozie, D. Tuscher, L. Nierves, J. Tsui, P. F. Huesgen and P. F. Lange (2019). "Sensitive Determination of Proteolytic Proteoforms in Limited Microscale Proteome Samples." Mol Cell Proteomics **18**(11): 2335-2347.
